# Case report: Fatal poisoning caused by additive effects of two barbiturates

**DOI:** 10.3389/fphar.2023.1196565

**Published:** 2023-05-24

**Authors:** Sella Takei, Hiroshi Kinoshita, Mostofa Jamal, Mitsuru Kumihashi, Tadayoshi Yamashita, Etsuko Tanaka, Sachiko Kawahara, Hiroko Abe, Kunihiko Tsutsui, Shoji Kimura

**Affiliations:** ^1^ Departments of Forensic Medicine, Faculty of Medicine, Kagawa University, Takamatsu, Japan; ^2^ Biodesign Inc, Tokyo, Japan; ^3^ Kagawa Prefectural University of Health Sciences, Takamatsu, Kagawa, Japan

**Keywords:** pentobarbital, phenobarbital, additive pharmacological effects, massive ingestion, forensic toxicology

## Abstract

A case of fatal poisoning involving multiple psychotropic drugs is presented. Quantitative toxicological analysis showed femoral blood concentrations of pentobarbital, phenobarbital, duloxetine, acetaminophen and tramadol were 10.39, 22.57, 0.22, 0.61 and 0.22 μg/ml, respectively. We concluded that the death was due to the additive effects of two barbiturates. As both pentobarbital and phenobarbital act on gamma-aminobutyric acid (GABA) receptors, central nervous system activity was suppressed, causing respiratory depression. Additive pharmacological effects should be considered in cases of massive ingestion of multiple drugs.

## 1 Introduction

Poisoning due to ingestion of multiple psychotropic drugs is sometimes observed. Barbiturates have been wide use in clinical practice since the early 20th century (Baselt, 2008). Pentobarbital is classified as a short-acting barbiturate, and is used as a sedative-hypnotic agent (Baselt, 2008). Phenobarbital is another barbiturate derivative, used as a sedative and anticonvulsant (Baselt, 2008). Both drugs are classified as barbiturates with pharmacological mechanisms involving the same target: the neuronal gamma-aminobutyric acid (GABA) receptor. As a result, both suppress neuronal activity (Charney et al., 2006). Although the incidence of poisoning with barbiturates has declined along with the decreased use as sedative-hypnotics, poisoning remains a problem (Charney et al., 2006). Here we report a case of death due to additive barbiturate toxicity in a case involving ingestion of multiple psychotropic drugs.

## 2 Case report

A male in his thirties was found dead in his house. Subsequent police investigations revealed that the deceased had been prescribed phramacotherapies for insomnia, psychosis and liver dysfunction. The patient had a history of intentional drug overdose. Medico-legal autopsy revealed slight contusions to the right elbow, but these were not considered contributory to the cause of death. No findings of natural disease were observed.

The deceased was 163 cm in height and weighed 67 kg. The heart weighed 409 g and contained 260 ml of blood without coagulum. The brain weighed 1,541 g and was slightly edematous. The left and right lungs weighed 499 and 791 g, respectively, and were congested. Histological examination revealed marked congestion and edema of the lungs. The other organs showed no notable changes other than congestion. The stomach contained 850 ml of brownish muddy liquid. A drug-screening test using a Triage^TM^ panel (Biosite Diagnostic, San Diego, CA, United States) yielded positive results for barbiturates and benzodiazepines. *Postmortem* samples such as bloods, urine, cerebrospinal fluid, bile and stomach contents were collected for toxicological investigations.

Toxicological analyses using liquid chromatography tandem mass spectrometry (LC-MS/MS) were performed as described previously (Kinoshita et al., 2017). In brief, liquid chromatography separations were carried out using an Ekspert^TM^ UltraLC 100-XL system (Eksigent part of Sciex, Framingham, MA, United States of America). An L-column2 ODS (1.5 mm × 150 mm; 5.0 µm particle size; Chemicals Evaluation and Research Institutes, Tokyo, Japan) was used with a mobile phase comprising solvent A (5% methanol containing 10 mM ammonium formate) and solvent B (95% methanol containing 10 mM ammonium formate) with a flow rate of 0.1 ml/min. A QTrap^®^ 4,500 tandem mass spectrometer (Sciex) was used to obtain mass spectra. Quantitation of ethanol was performed using headspace gas chromatography.

## 3 Results and discuccion

Toxicological analyses identified pentobarbital, phenobarbital, duloxetine, acetaminophen and tramadol in all specimens, and lorazepam was identified from urine alone. No ethanol was detected in the blood or urine. Table 1 shows quantities for each drug in samples from the victim. We also measured concentration of drugs from portal vein blood. Concentrations of pentobarbital and phenobarbital in the portal vein were higher than those in femoral venous blood, and concentrations of those drugs in stomach contents were markedly higher than concentrations of other drugs. The total doses of pentobarbital and phenobarbital remaining in stomach contents were 0.54 g and 1.38 g, respectively. Those results indicated that the deceased was in the absorption phase of drug pharmacokinetics at the time of death. Drug concentrations in portal vein blood may be useful for evaluating the interval following drug ingestion. As bile concentrations of drugs such as barbiturates, acetaminophen and duloxetine are also higher than other specimen sources, this site may be useful for drug screening (Bévalot et al., 2016).

**TABLE 1:** Concentrations found for each drug in post-mortem samples (µg/ml).

	left heart blood	right heart blood	femoral venous blood	blood in portal vein	urine	CSF	bile	stomach contents
Acetaminophen	0.50	0.41	0.61	0.85	36.93	0.33	18.77	0.24
duloxetine	0.80	0.23	0.22	0.33	0.06	0.02	1.25	0.63
lorazepam	BDL	BDL	BDL	BDL	0.06	BDL	BDL	BDL
pentobarbital	9.92	7.93	10.39	16.34	3.38	5.55	22.15	634.36
phenobarbital	23.27	16.95	22.57	30.69	21.73	11.95	38.83	1,631.97
tramadol	0.34	0.18	0.22	0.24	0.38	0.27	0.55	0.95

Table 2 indicates currently established fatal, toxic and therapeutic levels (Baselt, 2008; Schulz et al., 2012). Analytical results indicated concentrations of duloxetine slightly exceeding the therapeutic level, a toxic level of pentobarbital and a therapeutic level of phenobarbital, while acetaminophen and tramadol were both within normal therapeutic ranges in femoral venous blood. However, fatal concentration ranges of barbiturates are known to vary widely (Terada et al., 2005; Charney et al., 2006). As fatal poisonings with relatively low blood concentrations of pentobarbital or phenobarbital have been reported (Parker et al., 1970; Moffat et al., 2004), we have to consider that a narrow margin of safety exist for barbiturates. During poisoning, barbiturates cause loss of consciousness and respiratory arrest by depressing activity of the central nervous system (Charney et al., 2006). Congestion of the lungs may be a result of respiratory insufficiency.

**TABLE 2:** Therapeutic, toxic, and fatal ranges in blood in each drug (µg/ml).

	Therapeutic range^a^	Toxic range^a^	Lethal range^a^
Acetaminophen	5–25	100–150	90–387
duloxetine	0.03–0.12	0.24	0.91–2.5
lorazepam	0.02–0.25	0.3–0.5	0.52–2.8
pentobarbital	1–10	10–19	5–169
phenobarbital	10–40	30–40	55–116
tramadol	0.1–1	1	2–38.3

^a^ Therapeutic toxic and lethal ranges are cited from the reference (Baselt, 2008; Schulz et al., 2012).

Duloxetine is a synthetic serotonin and norepinephrine reuptake inhibitor used in the treatment of depression (Baselt, 2008). Since the blood concentration of duloxetine slightly exceeded the therapeutic level, but was not a toxic level, the contribution to his death seems likely to have been smaller. Acetaminophen is an analgesic agent and tramadol is a synthetic opioid receptor agonist used as a narcotic analgesic (Baselt, 2008). Concentration of both acetaminophen and tramadol were within or below therapeutic ranges, so those drugs were also considered to have contributed less to the victim’s death. Lorazepam, a derivative of benzodiazepine, is prescribed as an anxiolytic (Baselt, 2008). As the blood concentration was below the limit of detection, lorazepam seems to have made no contribution to his death.

Using the toxicokinetic parameters of pentobarbital and phenobarbital, such as distribution volume (0.5–1.0 and 0.5–0.6 L/kg, respectively) (Baselt, 2008), the body weight of the victim and femoral blood levels, we estimated the total amounts of pentobarbital and phenobarbital ingested. The estimated doses of pentobarbital and phenobarbital calculated from toxicokinetic parameters were 0.35 and 0.76 g, respectively. The amount of pentobarbital and phenobarbital remaining in the stomach (volume, 850 ml) were approximately 0.54 and 1.38 g, respectively. The estimated total doses ingested were thus >0.89 g of pentobarbital and >2.14 g of phenobarbital, based on toxicokinetic estimations. As the minimum lethal doses of pentobarbital and phenobarbital have been reported as 1 and 1.5 g, respectively (Moffat et al., 2004; Terada et al., 2005), these drugs seem likely to have contributed to the present fatal results via CNS depression.

Based on the autopsy findings and toxicological examination results, we concluded that multiple drug ingestion led to this death, mainly due to the additive toxicity of pentobarbital and phenobarbital via GABA receptor. The combination with pentobarbital and phenobarbital may have additively potentiated CNS depression in this case.

## 4 Brief summary

Pentobarbital and phenobarbital are barbiturates targeting the neuronal gamma-aminobutyric acid (GABA) receptor, which supress the activity of central nervous system. Here we report a case of poisoning involving multiple psychotropic drugs. Quantitative toxicological analysis revealed femoral blood concentrations of pentobarbital and phenobarbital to be 10.39 and 22.57 μg/ml, respectively. We concluded that the death was due to the additive effects of two barbiturates. Additive pharmacological effects should be considered in cases of massive ingestion of multiple drugs.

## Data Availability

The original contributions presented in the study are included in the article/supplementary material, further inquiries can be directed to the corresponding author.

## References

[B1] Baselt R. C. (2008). Disposition of toxic drugs and chemicals in man. 8th ed. Foster City, CA: Biochemical Publications.

[B2] Bévalot F. Cartiser N. Bottinelli C. Guitton J. Fanton L. (2016). State of the art in bile analysis in forensic toxicology. Forensic Sci. Int 259, 133–154. 10.1016/j.forsciint.2015.10.034 26773224

[B3] Charney D. S. Mihic S. J. Harris R. A. (2006). “Hypnotics and sedatives,” in Goodman and Gilman’s the pharmacological basis of therapeutics. Editors Brunton L. L. Lazo J. S. Parker K. L. (New York, Chicago: McGraw-Hill), pp401–427.

[B4] Kinoshita H. Tanaka N. Takakura A. Kumihashi M. Jamal M. Ito A. (2017). Flunitrazepam in stomach contents may be a good indicator of its massive ingestion. Rom. J. Leg. Med. 25, 193–195. 10.4323/rjlm.2017.193

[B5] Moffat A. C. Osselton M. D. Widdop B. (2004). “Toluene,” in Clark's analysis of drug and poisons. 3rd edition (London, Chicago: Pharmaceutical Press).

[B6] Parker K. D. Elliott H. W. Wright J. A. Nomof N. Hine C. H. (1970). Blood and urine concentrations of subjects receiving barbiturates, meprobamate, glutethimide, or diphenylhydantoin. Clin. Toxicol. 3, 131–145. 10.3109/15563657008990108 5520387

[B7] Schulz M. Iwersen-Bergmann S. Andresen H. Schmoldt A. (2012). Therapeutic and toxic blood concentrations of nearly 1000 drugs and other xenobiotics. Crit. Care 16, R136. 10.1186/cc11441 22835221 PMC3580721

[B8] Terada M. Watanabe R. (2005). “Barbiturates,” in Drugs and poisons in humans, a handbook of practical analysis. Editors Suzuki O. Watanabe K. (Berlin, Heidelberg: Springer-Verlag), pp301–313.

